# High burden of wasting among children under-five with hydrocephalus receiving care at CURE children’s hospital in Uganda: a cross-sectional study

**DOI:** 10.1186/s40795-024-00819-z

**Published:** 2024-01-17

**Authors:** Naula Grace, Edith Mbabazi, David Mukunya, Josephine Tumuhamye, Humphrey Okechi, Emmanuel Wegoye, Peter Olupot-Olupot, Joseph KB Matovu, Leah Hopp, Agnes Napyo

**Affiliations:** 1 Department of Community and Public Health, Busitema University Faculty of Health Sciences, Mbale, Uganda; 2 Department of Medicine and Research, Cure Children’s Hospital, Mbale, Uganda; 3 Department of Disease Control and Environmental Health, Makerere University School of Public Health, Kampala, Uganda; 4 Department of Community Health, Akisyon a Yesu Presbyterian Clinic, Nakaale, Karamoja Uganda; 5 Department of Nursing Sciences, School of Medicine, Kabale University, Kabale, Uganda; 6 Department of Epidemiology and Biostatistics, School of Public Health, Makerere University, Kampala, Uganda; 7 Mbale Clinical Research Institute, Mbale, Uganda

**Keywords:** Children under-five, Hydrocephalus, Nutritional status, Wasting, Nutrition

## Abstract

**Background:**

Hydrocephalus is one of the most common neurological disabilities presenting in children. Although there are limited studies on its association with wasting, neurological comorbidities such as dysphagia have been associated with an increased risk of wasting in children. In this study, we aimed to determine the prevalence and factors associated with wasting in children less than five years with hydrocephalus.

**Methods:**

We conducted a cross-sectional study at various satellite clinics of CURE Children’s Hospital in Uganda between September and November 2021. Children with hydrocephalus were identified at the outpatient departments of the satellite clinics of the Cure Children’s Hospital and these include Mbale, Gulu, Lira, Jinja and Katalemwa. A structured questionnaire was used to collect information on several variables including (1) for the mother: socio-demographic characteristics, partner support, and wealth index (2) for the child: socio-demographic characteristics, clinical symptoms, feeding difficulties and neural comorbidity. Anthropometric measurements were also taken and these included the mid-upper arm circumference. Data were analysed using Stata version 14. We estimated adjusted odds ratios and their corresponding 95% confidence intervals while relying on multivariable logistic regression models.

**Results:**

The prevalence of wasting among children with hydrocephalus was 23.2% (*n* = 89/384) (95%CI: 19 − 27.7%). Their mean age was 19.5 months (SD 16.8). Most of the children were below 12 months (47.9%) and were male (57.5%). The factors associated with wasting among children with hydrocephalus included: having; difficulty in chewing and swallowing (AOR = 2.6, (95%CI:1.05–3.94), a poor appetite (AOR = 1.74, (95%CI: 1.31–2.32), difficulty in breathing (AOR = 1.9, (95%CI: 1.18–3.16), chocking on food (AOR = 1.42, (95%CI:1.1–1.9) and attending the Mbale satellite clinic (AOR = 2.1 (95% CI 1.19–3.7). Children under 5 years of age with hydrocephalus that were born to women whose highest level of education was 7 to 10 years of formal schooling (AOR = 0.32, 95%CI: (0.12–0.87) were less likely to be wasted.

**Conclusions and recommendations:**

The prevalence of wasting among children with hydrocephalus was high. The factors associated with wasting were mainly feeding challenges. We recommend that children with hydrocephalus should be given greater attention regarding their nutrition especially those with various forms of feeding difficulties. The caregivers of children with hydrocephalus should receive counseling on nutrition and on the best modalities to rely on while feeding their children.

## Introduction

Hydrocephalus is characterized by the expansion of the cerebral ventricles due to an abnormal accumulation of cerebrospinal fluid which can occur in conjunction with, or in absence of, changes to intracranial pressure. Infants commonly present with progressive enlargement of the head whereas children older than 2 years generally present with signs and symptoms of intracranial hypertension [1]. Hydrocephalus not only has a genetic predisposition, but can also be acquired. Congenital hydrocephalus has been linked to genes that regulate brain growth and development. Hydrocephalus can also be acquired, mostly from pathological processes that affect ventricular outflow, subarachnoid space function, or cerebral venous compliance [1].

While the global health burden of infant hydrocephalus is substantial for rich and poor countries alike, it is especially conspicuous in sub-Saharan Africa (SSA), where approximately 180,000 new cases arise each year [2, 3]. In low- and middle-income countries (LMICs), post-infectious hydrocephalus (PIH) following neonatal sepsis is the most common form of infant hydrocephalus [4]. Regardless of aetiology, prompt surgical intervention prevents dire outcomes and leads to more favourable results for children [5]. Treatment options include shunt and endoscopic approaches, which should be individualised to each child [6, 7].

A number of risk factors for hydrocephalus in children have been documented and these include: (1) child-related factors including: being born premature [8, 9], infections such as ventriculitis and meningitis [4]. (2) Maternal factors including: age, low socioeconomic status, family history, trauma during pregnancy, lack of prenatal care, multiparous gestation, common cold with fever, ischemic heart disease, thyroid disease diabetes, chronic hypertension, gestational hypertension, alcohol use during pregnancy, prenatal exposure to medications like tribenoside, metronidazole, anaesthesia, opioids [8–10], (3) lifestyle modifiable maternal factors such as obesity, high-density lipoprotein (HDL) cholesterol [8, 9] and (4) environmental factors such as altitude and paternal occupation [9].

These children can also get complications arising from hydrocephalus and these include: shunt infections, death arising from a blocked shunt, blindness, endocarditis, arachnoiditis, renal and heart failure [11].

The association between hydrocephalus and under nutrition has been documented [12, 13]. In one study it was found that under nutrition increased the development of hydrocephalus among children with tuberculous meningitis [13]. In another study, under nutrition was associated with complications after shunt surgery and these include shunt infection, shunt revision and mortality [14].

In an attempt to streamline some nutrition-related concepts, malnutrition refers to deficiencies or excesses in nutrient intake, imbalance of essential nutrients or impaired nutrient utilization. Malnutrition is double-barrelled consisting of both under nutrition and overweight and obesity. Under nutrition manifests in four broad forms including: wasting, stunting, underweight, and micronutrient deficiencies. Wasting is defined as low weight-for-height. It often indicates recent and severe weight loss, although it can also persist for a long time. It usually occurs when a person has not had food of adequate quality and quantity and/or they have had frequent or prolonged illnesses [15].

Children with hydrocephalus often have imbalanced energy intake, gastrointestinal problems and therefore are susceptible to nutritional challenges leading to wasting [14, 16, 17]. Evidence demonstrates that malnutrition in children with hydrocephalus can be as high as 31.4% [18]. Wasting is associated with factors such as feeding challenges [19], infections [20] and socio-economic status of the caregivers [21]. Wasting in children is also associated with a higher risk of death if not treated properly [15]. Children with hydrocephalus that are wasted are susceptible to increased risk of shunt infection, failure to thrive, dysfunctional metabolic profile, poor wound healing, increased infections, impaired immunity which delays the recovery process and increases health care costs [18, 22–25].

The nutritional status of children with hydrocephalus not only has an impact on their health and development but also on their overall survival [11–13, 18]. Therefore, the nutritional status of children with hydrocephalus should be evaluated to enable the alignment of appropriate measures that can prevent it. Various methods have been used to assess the nutritional status in paediatric populations. However, for paediatric populations with hydrocephalus, these methods vary including measuring weight for length and the mid upper arm circumference. One study has found that there is no significant difference between relying on MUAC or WFL for the measurement of nutritional status in macrocephalic pediatric populations [26]. For purposes of this paper, we rely on MUAC to measure wasting among children under 5 with hydrocephalus and to determine the factors associated with wasting.

## Methods

### Study design and setting

We conducted a cross-sectional study at CURE Children’s Hospital in Uganda (CCHU) from September to November 2021. This hospital is among the leading paediatric hospitals for the treatment of neurological conditions and brain surgery. The hospital also provides minimally-invasive endoscopic neurosurgical procedures. The neurosurgical procedures are done for conditions like spina bifida, brain tumours, hydrocephalus and other neural tube defects. The main centre for the hospital is located at Mbale city. This location also acts as a teaching hospital which consists of three operating rooms, an 18-bed intensive care unit and 37 ward beds. During the time of the study, the hospital was operating various satellite clinics in Gulu, Lira, Jinja and Katalemwa. Each of the satellite clinics is held once a month. The clinic staff that work at CCHU in Mbale City are the ones that operate these clinics. These outpatient clinics are held to reduce on transport costs for the patients that come from very distant places and also to decongest the CCHU at the Mbale site. The Mbale site operates on a daily basis because it is a referral site for all the other satellite clinics and it is an inpatient site in the event that admissions or specialized attention and care are required. All the five satellite clinics are collectively referred to as CCHU. In this study, we included participants that sought care from Mbale, Gulu, Lira, Jinja and Katalemwa clinics.

CCHU serves a wide range of catchment population. Patients travel from all over Uganda and beyond to seek neural services from CCHU. The services at CCHU are organised in departments and these are offered by neurosurgeons, nurses, laboratory technologists, physiotherapists, radiologists, anaesthesiologists, medical officers, counsellors, clinical officers.

### Participants

We recruited participants from five satellite clinics and these included Mbale, Gulu, Lira, Jinja and Katalemwa. The study included children below the age of five with hydrocephalus receiving treatment at CCHU and those whose mothers had consented to their participation. Children with a diagnosis other than hydrocephalus were excluded. We confirmed hydrocephalus with the help of a CT scan that had been done as routine imaging at CCHU. The diagnosis for hydrocephalus is made at the main referral site in Mbale and documented in the patient’s file. The patient files are routinely taken to the satellite clinics to be used during patients’ reviews. We were able to access the patient’s files to confirm the diagnosis of hydrocephalus. We did not document the date or timing of diagnosis of the participant.

### Data collection procedures

Prior to the start of the study, the study team (which included the researcher and two trained, qualified research assistants) introduced the study and its procedures to the clinic staff at each satellite clinic. The research assistants had prior experience in data collection and taking anthropometric measurements in paediatric populations. The research assistants were further trained on the study procedures prior to commencement of the study. When the mother and her child arrived at any of the satellite clinics, they begun at the outpatient department where they were triaged by a clinic staff. During triage, anthropometric measurements including mid-upper arm circumference, weight and height were taken. It is during triage that the clinic staff did the screening to identify the eligible child. If found to be eligible, the clinic staff mainly a nurse, referred the mother-baby pair to the study team to confirm eligibility and initiate the informed consenting process. Mother-baby pairs were included in the study. The caregiver was considered to be a mother, father or other relative that took responsibility over the infant. In this study, all the caregivers were actually the mothers to the infants. The consenting process was done with the mothers to the infants. Once the mother had consented to the inclusion of her child into the study, the research assistant then administered a questionnaire in the form of an interview where the research assistant asked the mother questions to which the she responded and these responses were written down on the paper-based questionnaire. Each interview lasted a maximum of 20 to 30 min. The questionnaire used was specifically developed for this study and has been attached as a supplementary file. We tried as much as possible not to interfere with the normal clinic flow. The children were consecutively enrolled on the study until the desired sample size was reached.

The triage was done to establish severity of the condition and also to check if they are to see a social worker or clinician. After triage, consenting and going through the interview, the mother-baby pair was then sent to see the respective care provider to continue with the routine clinic flow. Those that were from the clinician and needed further investigation were sent to the laboratory. Depending on the laboratory or imaging results they were either sent for admission in a clinical setting, back home, or referred.

### Sampling and sample size

The sample size was calculated by using the Cochrane formula for single population proportion: n = Z^2^ *p*q/e^2^ we took the prevalence of malnutrition among children with hydrocephalus to be 31.4% [18] at a 95% CI with a 5% margin of error. For this formula, z is the selected critical value of desired confidence level at 1.96, p is the estimated prevalence of malnutrition among children with hydrocephalus, q = (1– p) and e is the desired level of precision. While factoring in a 10% non-response, we estimated a sample size of 368 participants. We, however, included 384 participants that we enrolled consecutively till we arrived at the estimated sample size. We controlled for clustering at analysis.

### Measurements

#### Outcome variable

The outcome of interest for this study was wasting indicated by the mid-upper arm circumference (MUAC) for children under 5 years with hydrocephalus [26]. The MUAC measurement was taken from a non-dominant arm between the elbow and the shoulder. For each child, the nurse bent the arm at a 90^0^. She then found the top of the shoulder and the tip of the elbow and placed the MUAC tape at the top of the shoulder while keeping it at eye level. She then put her right thumb on the tape where it meets the tip of the elbow (the endpoint). She found the middle of the upper arm by carefully folding the endpoint to the edge of the tape. She then put her thumb on the point where the tape was folded (midpoint). The midpoint was then marked with an erasable pen. The arm was then straightened and the tape wrapped firmly around at the marked midpoint by adjusting it through the tape window while taking care not to have it too loosely or too tightly. The measurement was then read off in centimetres at the point where the arrow points inward. Children that had a MUAC reading of less than 12.5 cm were classified as being ‘wasted’ and those that had 12.5 cm or more were ‘not wasted’.

Wasting in children under 5 years is considered to be the weight of the child relative to their height [24]. However, in our case, we did not consider weight in the computation of wasting because for children with hydrocephalus, weight would not be an objective score to rely on since the accumulation of the cerebrospinal fluid in the brain which would contribute to it. This is why we relied on the mid-upper arm circumference to compute the wasting. A study done to compare the between the use of MUAC and weight-for-length for the measurement of wasting among macrocephalic pediatric populations found that there was no statistical difference in the results got from using the two methods [26].

#### Explanatory variables

The mothers were asked questions on their own socio-demographic characteristics and those of their child as well as questions on issues surrounding the child’s feeding, child’s health status and presence of comorbidities. We also took anthropometric measurements (height / length, weight and mid-upper arm circumference).

For the child: we asked about the age in completed number of months, date of birth to confirm age and sex of the child. We also asked the mother if the child had suffered any of the following in the seven days prior to the date of the interview: fever, diarrhoea, vomiting, cough, difficulty in breathing or constipation. We also asked the mother on issues surrounding the feeding of their child. These issues included: difficulty in eating, difficulty in swallowing, difficulty in chewing food and vomiting during and after meals. We checked the child’s record to ascertain if they had any neural comorbidity including Spina bifida, cerebral palsy or epilepsy.

For the mother: we asked each of them about their age in completed number of years, marital status (if they were married, separated or single), education level (as the number of years that they had attended formal education), number of children below the age of 5 years (we categorised this as < 3 and ≥ 3 children), employment status (categorised as ‘formally employed’, ‘self-employed’ or ‘not employed’). Partner support was assessed by asking mother if she was escorted to hospital by the partner or if the partner helped in paying hospital bills. We asked about some socio-economic factors such as source of food at home, source of light at home and source of water used at home. We created a composite index of wealth (socio-economic status) using principle component analysis (PCA). We used PCA on source of food at home, source of light at home and source of water used at home. Scores were obtained and categorized into three groups (terciles) ranging from the lowest (poorest) to the highest (least poor).

### Anthropometric measurements

Measurements of mid-upper arm circumference, weight and height were taken for the children under 5 years of age with hydrocephalus. We have already described how MUAC was measured. The height and weight of the children were measured while standing for children who could stand and those unable to stand, height was taken while lying and weight was measured using an infant weighing scale. WHZ scores were calibrated using the WHO standard growth charts. The anthropometric measurements included in this study are routinely done at every clinic visit for these children. Anthropometric measurements were taken by a well-trained clinic nurse during triage at Cure children’s hospital and all the satellite clinics. We documented these readings at the point at which they had been measured by the triage nurse.

### Data collection tools

Data were collected using a structured and pre-tested questionnaire. This was an interviewer-administered questionnaire. It was administered by well-trained and qualified research assistants. This questionnaire included questions on socio-demographic characteristics of both the mother and her child as well as questions on issues surrounding the child’s feeding, child’s health status and presence of comorbidities. There was a section in which anthropometric measurements (height / length, weight and mid-upper arm circumference) were recorded. Each interview lasted a maximum of 30 min.

### Data management and analysis

Data were checked for completeness, coded and doubly entered into Microsoft Excel. Data were then exported to STATA version 14.0 (Stata Corp, College Station, Texas, U.S.A.) for analysis. Continuous data, if normally distributed, were summarised into means and standard deviations and if skewed, were summarised into medians with their corresponding interquartile ranges (IQR). Categorical variables were summarised into frequencies and percentages. The prevalence of wasting among children under 5 years with hydrocephalus was estimated and its 95% confidence limits calculated using the exact method. Associations and correlations of the variables were computed using the Chi-square. Bivariate and multivariable analysis was done using logistic regression analysis. All variables that had a p-value < 0.25 at bivariable analysis and those of biological plausibility were collectively put into a multivariable model to control for confounding. We checked for confounding by calculating the percentage change in each effect measure by removing or introducing one variable at a time. If a variable caused more than 10% change in any effect measure then it was considered a confounder. We estimated unadjusted and adjusted odds ratios (OR) with their corresponding 95% confidence intervals to identify the factors associated with child wasting. We controlled for clustering at the level of the satellite clinics from which data was obtained, to cater for heterogeneity amongst participants in these 5 different data collection points. For this study, we did not compare between the use of MUAC and weight-for-length for the measurement of wasting because it was not the intended objective. As such we never embarked on any sensitivity analyses.

## Results

### Characteristics of the children who are under five with hydrocephalus

We enrolled 384 children whose mean age was 19.5 months (SD 16.8). The majority of these children were male (57.5%), below 12 months (47.9%) and receiving their neural care from Mbale Cure Children’s Hospital (35.4%). A lesser proportion of the children had no difficulty in chewing and swallowing food (16.4%), vomited during meals (21.9%), had poor appetite (17.2%), and choked on food (32%). Fewer children experienced difficulty in breathing (9.6%), had had diarrhea (13.8%) and constipation (6.8%). Nineteen [19] percent had spina bifida. A few children had cerebral palsy (2.3%) (Table 1).

### Characteristics of mothers to the children who are under five with hydrocephalus

The mean age of the mothers was 27.6 years (SD 6.8). Majority of the mothers were married (78.6%), had attained at least a primary education (53.9%) and had less than 3 living children below the age of 5 years (76.8%). The biggest proportion of mothers were unemployed (65.9%) and belonged to the lowest wealth stratum (43.5%). Many of these mothers (88%) were not accompanied by their spouses to the hospital and for 50.8%, financial support in form of transport facilitation to the hospital was not given by the spouse.

### Prevalence of wasting among children less than 5 years with hydrocephalus

The prevalence of wasting among children under 5 years with hydrocephalus was 23.2%, n = 89 / 384 (95%CI: 19 − 27.7%) (Table 1).

**Table 1:** Characteristics of children with hydrocephalus and their mothers

Characteristics	Total Number	Wasted	Not Wasted	P-Value
	*N* = 384	*n* = 89 (23.2%)	*n* = 295 (76.8%)	
		n (%)	n (%)	
**Characteristics of the children**				
**Age (in months)**				0.093
0–12	184 (47.9)	56 (30.4)	128 (69.6)	
13–24	85 (22.1)	18 (21.2)	67 (78.8)	
25–36	46 (11.98)	5 (10.9)	41 (89.1)	
37–48	36 (9.38)	4 (11.1)	32 (88.9)	
49–59	33 (8.59)	6 (18.2)	27 (81.8)	
**Sex**				0.414
Female	163 (42.5)	42 (25.8)	121 (74.2)	
Male	221 (57.5)	47 (21.3)	174 (78.7)	
**Height (in cm)**				
48–68.9	116 (30.2)	51 (44)	65 (56)	0.000
69–98.9	245 (63.8)	37 (15.1)	208 (84.9)	
99–112	23 (6)	1 (4.3)	22 (95.7)	
**Weight (in kg)**				
2.5–8.4	147 (38.3)	75 (51)	72 (49)	0.000
8.5–14.4	197 (51.3)	14 (7.1)	183 (92.9)	
14.5–20.4	38 (9.9)	0 (0)	38 (100)	
20.5–24.4	2 (0.5)	0 (0)	2 (100)	
**Residence**				0.1428
Rural	277 (72.1)	74 (26.7)	203 (73.3)	
Urban	107 (27.9)	15 (14)	92 (86)	
**Clinic attended**				0.0009
Gulu	35 (9.1)	11 (31.4)	24 (68.6)	
Jinja	51 (13.3)	7 (13.7)	44 (86.3)	
Kampala	84 (21.9)	11 (13.1)	73 (86.9)	
Lira	78 (20.3)	14 (17.9)	64 (82.1)	
Mbale	136 (35.4)	46 (33.8)	90 (66.2)	
**Child has difficulty in chewing and swallowing**				0.0008
No	321 (83.6)	62 (19.3)	259(80.7)	
Yes	63 (16.4)	27 (42.9)	36 (57.1)	
**Child has difficulty in breathing**				0.0003
No	347 (90.4)	72 (20.8)	275 (79.2)	
Yes	37 (9.6)	17 (45.9)	20 (54.1)	
**Child vomits during meals**				0.0048
No	300 (78.1)	58 (19.3)	242(80.7)	
Yes	84 (21.9)	31(36.9)	53(63.1)	
**Child has a good appetite**				0.0000
No	66 (17.2)	25 (37.9)	41 (62.1)	
Yes	318 (82.8)	64 (20.1)	254 (79.9)	
**Child chocks on food**				0.0000
No	261(68)	44(16.9)	217 (83.1)	
Yes	123 (32)	45(36.6)	78 (63.4)	
**Child has cough**				0.8622
No	244 (63.5)	56 (23)	188 (77)	
Yes	140 (36.5)	33 (23.6)	107 (76.4)	
**Child has diarrhea**				0.7963
No	331(86.2)	76 (23)	255 (77)	
Yes	53 (13.8)	13 (24.5)	40 (75.5)	
**Child has constipation**				0.6842
No	358 (93.2)	84 (23.5)	274 (76.5)	
Yes	26 (6.8)	5 (19.2)	21 (80.8)	
**Child has spina bifida**				0.9838
No	311 (81)	72 (23.1)	239 (76.9)	
Yes	73 (19)	17 (23.3)	56 (76.7)	
**Child has cerebral palsy**				0.438
No	375 (97.67)	86 (22.9)	289 (77.1)	
Yes	9 (2.3)	3 (33.3)	6 (66.7)	
**Maternal characteristics**				
**Age (in years)**				0.414
17–19	34 (8.9)	9 (26.5)	25 (73.5)	
20–29	214 (55.7)	48 (22.4)	166 (77.6)	
30–39	110 (28.6)	23 (20.9)	87 (79.1)	
40–49	26 (6.8)	9 (34.6)	17 (65.4)	
**Marital status**				0.8115
Married	302 (78.6)	72 (23.8)	230 (76.2)	
Separated	46 (12)	10 (21.7)	36 (78.3)	
Single	36 (9.4)	7 (19.4)	29 (80.6)	
**Educational level (completed years of education)**				0.0718
5 to 6 years	19 (5)	9 (47.4)	10 (52.6)	
7 to 10 years	207 (53.9)	51 (24.6)	156 (75.4)	
11 to 13 years	106 (27.6)	22 (20.8)	84 (79.2)	
> 13 years	52 (13.5)	7 (13.5)	45 (86.5)	
**No of children below 5 years**				0.0001
< 3 children	295 (76.8)	35 (11.9)	260 (88.1)	
≥ 3 children	89 (23.2)	24 (24.8)	65 (74.2)	
**Employment status**				0.0007
Formally employed	38 (9.9)	5 (13.2)	33 (86.8)	
Self employed	93 (24.2)	16 (17.2)	77 (82.8)	
Unemployed	253 (65.9)	68 (26.9)	185 (73.1)	
**Wealth index terciles**				0.0000
Lowest tercile	167 (43.5)	46 (27.5)	121 (72.5)	
Middle tercile	97 (25.3)	26 (26.8)	71 (73.2)	
Highest tercile	120 (31.2)	17 (14.2)	103 (85.8)	
**Accompanied to the facility by spouse**				0.4614
No	338 (88)	77 (22.8)	261 (77.2)	
Yes	46 (12)	12 (26.1)	34 (73.9)	
**Transport paid for by spouse**				0.8623
No	195 (50.8)	46 (23.6)	149 (76.4)	
Yes	189 (49.2)	43 (22.8)	146 (77.2)	

### Factors associated with wasting among children under 5 years with hydrocephalus

Mothers who had attained 7 to 10 years of formal schooling (AOR = 0.32 95%CI: 0.12–0.87) were less likely to have children that are wasted compared to their counterparts that had attained 5 to 6 years of formal schooling. Children that had any form of feeding challenge were more likely to be wasted compared to those who did not. These included having: difficulty in chewing and swallowing (AOR = 2.6, 95%CI: 1.05–3.94), poor appetite (AOR = 1.74, 95%CI: 1.31–2.32), difficulty in breathing (AOR = 1.93, 95%CI: 1.18–3.16) and chocking on food (AOR = 1.42, 95%CI: 1.04–1.95). Children attending the Mbale satellite clinic were more likely to be wasted (AOR = 2.1, 95%CI: 1.19–3.7) (Table 2).

**Table 2:** Factors associated with wasting among children under 5 years with hydrocephalus

Variable	Unadjusted OR (95% CI)	Adjusted OR (95% CI)
**Age of Child (months)**		
0–12	1.97(0.83–4.69)	1.35 (0.39–4.62)
13–24	1.21(0.56–2.61)	0.89 (0.25–3.24)
25–36	0.55(0.15–1.97)	0.43 (0.1–1.86)
37–48	0.56(0.2–1.61)	0.37 (0.39–4.62)
49–59	1	1
**Educational level of mothers (in completed number of years)**		
5 to 6 years	1	1
7 to 10 years	0.36 (0.15–0.88)	**0.32 (0.12–0.87)**
11 to 13 years	0.29 (0.1–0.88)	0.36 (0.1–1.29)
> 13 years	0.17 (0.04–0.73)	0.21 (0.04–1.23)
**Household Wealth Quintile**		
Low	1	1
Middle	0.97 (0.72–1.31)	0.97 (0.72–1.31)
High	0.84 (0.43–1.63)	0.84 (0.43–1.63)
**Occupation of the mothers**		
Formally employed	1	1
Self employed	1.37 (0.56–3.35)	0.75 (0.41–1.39)
Unemployed	2.43 (1.35–4.35)	1.32 (0.56–3.11)
**No of children below the ages of 5 years**		
< 3 children	1	1
≥ 3 children	2.59 (1.62–4.13)	1.95 (0.97–3.9)
**Child has difficulty in chewing and swallowing**		
No	1	1
Yes	3.13 (1.61–6.1)	**2.6 (1.05–3.94)**
**Child vomits during meals**		
No	1	1
Yes	2.44 (1.31–4.53)	1.06 (0.66–1.71)
**Child has a good appetite**		
Yes	1	1
No	2.42 (1.69–3.47)	**1.74 (1.31–2.32)**
**Child has difficulty in breathing**		
No	1	1
Yes	3.25 (1.72–6.18)	**1.93 (1.18–3.16)**
**Child chocks on food**		
No	1	1
Yes	2.85 (1.84–4.41)	**1.42 (1.04–1.95)**
**Clinic attended**		
Gulu	1	1
Jinja	0.35 (0.12–1.01)	**0.64 (0.44–0.95)**
Kampala	0.33 (0.13–0.85)	**0.49 (0.38–0.64)**
Lira	0.48 (0.19–1.2)	**0.57 (0.44–0.74)**
Mbale	1.12 (0.5–2.5)	**2.1 (1.19–3.7)**

## Discussion

The prevalence of wasting was so high at 23.9%. This is higher than the prevalence of 4% for children who are under 5 years and are wasted in the general Ugandan population [27]. In one study done in India among children aged 0–18 months with intracranial infections, malnutrition was found among 48.1% of them [14]. Our prevalence was lower than the one stated in this study probably because our study focused on hydrocephalus while this study focused on intracranial infections in general. Our prevalence was comparable to that found in another study done in Brazil to establish the nutritional profile of children with hydrocephalus. In this study, 31.4% of the children were malnourished [18]. These proportions are comparable because our study looked at a similar population like the one of Brazil. Generally, most of the studies involving nutritional status of children with hydrocephalus included very few participants (commonly under 100). A number of studies have been done on nutritional status among children with neural tube defects in general. Many studies have also looked at malnutrition in general.

Our study found that children under-5 with hydrocephalus whose mothers had a formal education of 7 to 10 years were less likely to be wasted compared to those children whose mothers had received a formal education of 5 to 6 years. The education level of the mother has long been recognized as an important influencer of the nutritional status of a child due to a number of reasons, such as improved access to and use of information, improved self-confidence and decision-making power, better health and nutrition knowledge and practices [25]. A number of studies have demonstrated the association between maternal education and the nutrition status of the child. For instance, in one done in Mexico, there was improved height for age among children born to women who had completed a primary level education [28]. In another pooled analysis done for Demographic Health Surveys across 62 countries, it was found that maternal education was associated with childhood under nutrition [29].

We also found that children that had any form of feeding challenge were more likely to be wasted compared to those who did not. These included: difficulty in chewing and swallowing, poor appetite, difficulty in breathing and chocking on food. Children with hydrocephalus experience physical and physiological stress associated with increased intracranial pressure and this affects their appetite [30]. These findings collaborate with findings from other studies [19, 31]. Good appetite might mean great chances of nutrient availability for the hydrocephalus child, hence less chances of being wasted. A study done in Nepal, at Kathmandu Medical College Teaching Hospital found a relationship between wasting and acute respiratory infections [32]. This could possibly be attributed to the fact that children with breathing difficulties have insufficient oxygen, which has been found to be correlated with low ATP synthesis [33], hence body metabolic processes including growth do not progress optimally.

Findings from the study revealed that chocking on food was significantly associated with wasting in children with hydrocephalus. Children who chocked on food had increased odds of being wasted compared to those who did not. Children who chock on food in most cases also have difficulty in swallowing [34] and therefore have reduced intake of food nutrients including several nutrients such as Vitamin A, Vitamin E, Manganese, and Magnesium, which undermines the proper physiology of the body, hence leading to wasting [35, 36] These findings are similar to those by Lopes [37] who revealed that children who chocked on food were more likely to be under nourished and wasted.

Children attending Mbale satellite clinic were more likely to be wasted. The satellite clnic at Mbale is the main referral centre for the Cure Children’s Hospital for children with neural disabilities. Children that have advanced neural complications will already have had compromised nutritional status in terms of wasting and so on [38]. The children that are referred to Mbale satellite clinic are children that have complications and other ailments that may need specialised attention and cannot otherwise be got at other satellite clinics. It is quite reasonable that these children may present at the Mbale satellite clinic when they are already wasted secondary to other ailments that they suffer from.

### Strengths and limitations

Most studies done on this subject involve few participants mainly under 100. Our study has more participants and this could have contributed to its power. We only included children with hydrocephalus and therefore these findings may only generalizable to this population. We never asked about the breastfeeding patterns or immunization schedules of the children yet these may be potential exposures for the age group of children included in this study.

## Conclusions and recommendations

The prevalence of wasting among children with hydrocephalus was high. The factors associated to wasting were mainly feeding challenges. We recommend that children with hydrocephalus should be given greater attention regarding their nutrition especially those with various forms of feeding difficulties. The caregivers of children with hydrocephalus should receive counseling on nutrition and on the best modalities to rely on while feeding their children.

## Data Availability

The datasets used and/or analysed during the current study are available from the corresponding author on reasonable request.
